# Nano-hesperetin ameliorates 6-hydroxydopamine-induced behavioral deficits and oxidative damage by up-regulating gene expression of antioxidant enzymes 

**DOI:** 10.22038/AJP.2022.21532

**Published:** 2023

**Authors:** Akbar Hajizadeh Moghaddam, Sara Alizadeh, Monireh Nejadi, Seyed Reza Mokhtari Sangdehi, Mahboobeh Zare, Mojtaba Ranjbar

**Affiliations:** 1 *Department of Animal Sciences, Faculty of Basic Sciences, University of Mazandaran, Babolsar, Iran*; 2 *Faculty of Herbs, Amol University of Special Modern Technologies, Amol, Iran *; 3 *Faculty of Biotechnology, Amol University of Special Modern Technologies, Amol, Iran*

**Keywords:** Antioxidant enzymes, Behavioral disorders, Nano-hesperetin, Parkinson’s disease, 6-Hydroxydopamine

## Abstract

**Objective:**

Hesperetin (Hst) has shown several pharmacological effects. The efficacy of Hst is highly restricted *in vivo* due mainly to poor bioavailability. This investigation was intended to compare the influence of Hst and nano-Hst treatment on 6-hydroxydopamine (6-OHDA)-induced behavioral deficits and oxidative stress in rats.

**Materials and Methods:**

Forty-two Wistar male rats were equally assigned to 6 groups: control, 6-OHDA, Hst5, Hst10, nano-Hst5, and nano-Hst10. Treatment with Hst and nano-Hst was initiated 1 day after the intrastriatal injection of 6-OHDA and continued for 28 days. Behavioral deficits were evaluated using apomorphine-induced rotation test (AIRT), narrow beam test (NBT) and novel object recognition test (NORT), and the hippocampus and striatum were used to evaluate oxidative stress-related parameters.

**Results:**

The rats injected only with 6-OHDA showed learning and memory deficits but Hst and nano-Hst treatments improved it (p<0.001). Compared to the control group, a marked promotion in Malondialdehyde (MDA) levels along with a marked reduction in activities and gene expression of antioxidant enzymes and reduced glutathione (GSH) levels in the hippocampus and striatum were observed in the 6-OHDA group (p<0.01). However, administration of Hst and nano-Hst remarkably diminished MDA levels (p<0.01), and significantly increased the activities (p<0.01) and gene expression of antioxidant enzymes (p<0.05) and GSH levels (p<0.01) compared to the 6-OHDA group. In most parameters, nano-Hst has shown better therapeutic effects than Hst.

**Conclusion:**

Our findings reveal that Hst can be considered as a potential candidate for the treatment of neurodegenerative diseases and that nano-Hst may have better bioavailability.

## Introduction

Parkinson’s disease (PD) is a chronic and progressive neurodegenerative disorder that results mainly from progressive degeneration and death of substantia nigra neurons. Clinically, symptoms such as postural instability, rigidity and resting tremor are the main characteristics of PD (Baluchnejadmojarad et al., 2017). PD also affects multiple neuronal systems both centrally and peripherally, that are associated with nonmotor symptoms, including memory impairments, depression and cognitive decline. Although the exact cause of the disease remains to be identified, oxidative damage to dopaminergic neurons plays a fundamental role in the neurodegeneration and movement disorder seen in PD (He et al., 2018). 6-Hydroxydopamine (6-OHDA) is typically employed as a selective catecholaminergic neurotoxin and a hydroxylated analog of the natural neurotransmitter dopamine to create PD models *in vitro* and *in vivo*, pointing to the role of oxidative stress in the etiopathogenesis of PD (Hritcu et al., 2011). The basic premise is that 6-OHDA induces a severe degenerative process in the nigrostriatal system by producing reactive oxygen species (ROS) and mitochondrial dysfunctions in animal models (Haleagrahara et al., 2011). 6-OHDA is then rapidly oxidized to produce large amounts of ROS to decrease levels of antioxidant enzymes (Kuruvilla et al., 2013). Current medical treatments for PD could only offer partial symptomatic relief and have side effects in addition to poor efficacy in stopping the disease progression. Therefore, there is an urgent need for novel therapies that halt or reverse the neuronal damage hampering PD progression (Maitra et al., 2021). The current research has also indicated that dietary habits play a key role in neurodegeneration in PD, where deficiency of antioxidant components in the body enhances the risk of PD (Martínez-Boo, 2021).

Hesperetin (Hst) is a naturally occurring flavonoid from citrus fruits with potent antioxidant and anti-inflammatory effects. It has been well established that Hst promotes cellular antioxidant defense enzymes through radical scavenging activity and exerts a protective influence against 6-hydroxydopamine (6-OHDA)-induced neurotoxicity in rats (Kiasalari et al., 2016). Further evidence also demonstrated that Hst not only scavenges free radicals but also improves antioxidant cellular capacity by enhancing the expression of various antioxidant enzymes genes such as glutathione reductase (*GRx*), superoxide dismutase (*SOD*) and Catalase (*CAT*) via the ERK/Nrf2 pathway (Chen et al., 2010; Roohbakhsh et al., 2014). Despite various pharmacological activities, Hst has poor water solubility and poor absorption through the gastrointestinal tract, therefore, it is rapidly metabolized and has a short half-life (Zeng et al., 2021). In addition to the above, Hst cannot cross the blood-brain barriers (BBB), which is a major stumbling block for the central nervous system (CNS) therapeutics (Kakran et al., 2015). To improve the bioavailability of Hst, various approaches have been used in terms of formulations and route of administration (Fathi et al., 2013; Mary Lazer et al., 2018). Nanoparticles drug delivery systems (NPs) have recently developed as one of the leading strategies to ameliorate the bioavailability, absorption, and biodistribution as well as slowing down the rapid metabolism and systemic elimination of drugs (Mohanty et al., 2012). On the other hand, since NPs are very small in size, they provides excellent surface features. The particles with size less than 100 nm can deliver bioactive substances across a number of biological barriers (i.e. the intestinal mucosa and BBB) (Kakran et al., 2015).

Hence, in our previous studies, we synthesized nano-Hst and investigated its therapeutic effects on models of Alzheimer's disease (kheradmand et al., 2018) and autism (Khalaj et al., 2018), but the therapeutic role of nano-Hst in PD models has not been explored to date. Thus, this investigation was performed to find out the influences of the Hst and nano-Hst on behavioral deficits, expression of antioxidant genes and oxidative damage in a rat model of PD induced by 6-OHDA.

## Materials and Methods

**Preparation of nano-Hst**

We prepared nano-Hst as earlier explained (kheradmand et al., 2018). Briefly, ethanol (1 ml) was added to Hst (5 mg) to obtain a 5 mg/ml drug solution, and then the rapid addition of n-hexane as an anti-solvent, led to the formation of a nanosuspension. The volume ratio of solvent to antisolvent was 1:20. 

**Animals and treatment**

**Figure 1:**
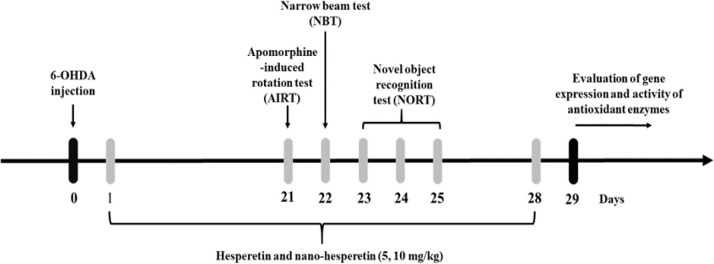
Experimental design

Wistar male rats (n=7 per group, 220–250 g) were purchased from Pasteur Institute of Iran, North Research Center (Amol, Iran). The animals were housed in a room with a temperature of 22 ± 1°C under a standard 12 hr light/dark cycle and fed with standard commercial food and water. The rats received Hst and nano-Hst at 5 and 10 mg/kg for 28 days. Since we administered different doses of Hst and nano-Hst in earlier investigations, in this study, lower doses of Hst were chosen to compare its therapeutic effects with nano-Hst. (Hajizadeh Moghaddam et al., 2020; Khalaj et al., 2018). Behavioral analyses were carried out from 10 a.m. to 15 p.m. in order to minimize circadian variations. One day after the last behavioral analysis, animals were deeply anesthetized with chloral hydrate (350 mg/kg, i.p.) and their hippocampus and striatum were removed for biochemical tests (Figure 1). The study was affirmed by the guidelines for the Care and Use of laboratory animals (IR.UMZ.REC.1396.007) and maintained in strict accordance with Ethical Committee for Animal Use of the University of Mazandaran. 

Forty-two rats were allocated into six groups: Group 1 (control): received 0.1% l-ascorbate saline for 28 days. Group 2 (6-OHDA): received a unilateral stereotaxic injection of 10 μg 6-OHDA (2 μl 0.1% l-ascorbate saline). Group 3 (Hst5): received 5 mg/kg of Hst daily for 28 days after 6-OHDA lesioning. Group 4 (Hst10): received 10 mg/kg of Hst daily for 28 days after 6-OHDA lesioning. Group 5 (nano-Hst5): received 5 mg/kg of nano-Hst daily for 28 days after 6-OHDA lesioning. Group 6 (nano-Hst10): received 10 mg/kg of nano-Hst daily for 28 days after 6-OHDA lesioning.

**6-OHDA lesion**

Animals underwent 6-OHDA surgery as described previously (Shrivastava et al., 2013). Chloral hydrate was used to anesthetize rats. After fixing them in the stereotaxic apparatus, rats were injected unilaterally with 10 μg 6-OHDA (2 μl of physiological saline containing 0.02% ascorbic acid) using a 22-gauge Hamilton syringe into the right striatum. The stereotaxic coordinates were: AP +1.0, ML +2.5, and DV +4.5 relative to bregma in mm, corresponding to the atlas of (Paxinos and Watson, 2006).

**Behavioral studies**

**Apomorphine-induced rotation test (AIRT)**

Rats were put into Plexiglas cylindrical cages (38 cm height and 28 cm diameter) and a number of complete rotations (ipsilateral and contralateral) were recorded at 10-min intervals for 1 hr. The net rotation count was considered the contralateral scores minus the ipsilateral ones (Fine et al., 2020). 

**Narrow beam test (NBT)**

NBT was conducted according to prior researches (Kiasalari et al., 2016). NBT consisted of a 1-m long, 2-cm thick, 30-mm height wooden beam. The total duration of passing over the beam was documented. The cut-off time on the rotarod was 160 sec. A fall was considered the maximum amount of time. Each animal was placed on the beam 5 times with a time interval of 5 min and tested. 

**Novel object recognition test (NORT)**

The rats were subjected to NORT as described previously (Hajizadeh Moghaddam et al., 2021). The discrimination ratio was measured as the detection time of novel object/the total time spent detecting the two objects multiplied by a percentage.

**Biochemical studies: Preparation of tissue homogenate**

All rats were decapitated after the last behavioral test and the brains were removed quickly. The hippocampus and striatum tissues were rapidly isolated and kept at −80°C. Tissue samples (150 to 200 mg of hippocampus and striatum tissue) were homogenized using a tissue homogenizer immersed in Tris-sucrose buffer (0.32 M sucrose in 10 nM Tris HCl buffer containing 1 mM EDTA (pH 7.4) and centrifuged at 12,000 rpm for 30 min at 4°C. Later on, the supernatant was stored (-80°C) for the biochemical estimation of oxidative stress markers. Bovine serum albumin was utilized as a standard to measure the protein level of the hippocampal and striatal homogenates according to Bradford's assay (Bradford, 1976). 

**Determination of oxidative stress markers**

Decline in GSH levels was evaluated according to (FUKUZAWA and TOKUMURAI, 1976). Malondialdehyde (MDA) levels were estimated by the method of (Esterbauer and Cheeseman, 1990). SOD and CAT enzymes activities were estimated quantitatively by a previously described technique (Genet et al., 2002). The Pinto and Bartley method was used for measurement of glutathione reductase (GRx) activity (Pinto and Bartley, 1969). 

**Quantitative real-time PCR analysis**

The mRNA expression levels were estimated by RT-qPCR as noted earlier (Moghaddam et al., 2020). To measure mRNA levels of *SOD*, *CAT* and *GRx* genes, total RNA from hippocampus and striatum tissues samples was extracted by RNeasy Mini Kit (QIAGEN) following the manufacturer’s instructions. The cDNA synthesis was performed using Superscript III Reverse Transcriptase (Fermentas) following the instructions of the manufacturer. Table 1 displays the sequence of the primers of the three studied genes. RNA levels were analyzed as specific RNA-to-GADPH ratios to determine the relative amounts of target genes.

**Table 1:** Sequences of primers used in qRT-PCR

Gene	Primer	Sequence	Amplicon lengths (bp)
*GAPDH*	forward reverse	5´-ATCCTGCACCACCAACTGC-3´ 5´-ACGCCACAGCTTTCCAGAG-3´	129
*CAT*	forward reverse	5´-GCGGATTCCTGAGAGAGTGG-3´ 5´-TCCAGCGACTGTGGAGAATCG-3´	154
*SOD*	forward reverse	5´-GACGAAGGGAGGTGGATGC-3´ 5´- GCCCTCCAGACTGAAATAGGC-3´	155
*GRx*	forward reverse	5´-GCCTTCACCCCGATGTATCAC-3´ 5´-GCATCTCATCGCAGCCAATC-3´	127

**Statistical analysis**

All results are expressed as mean±SD and P values below 0.05 were described significant. The Shapiro–Wilk was utilized to verify data normality. One-way ANOVA and then Tukey's multiple comparisons were utilized as a *post hoc* test using SPSS software to analyze difference of mean values among the groups.

## Results

**Effect of Hst and nano-Hst on rotational behavior induced by 6-OHDA in AIRT**

**Figure 2:**
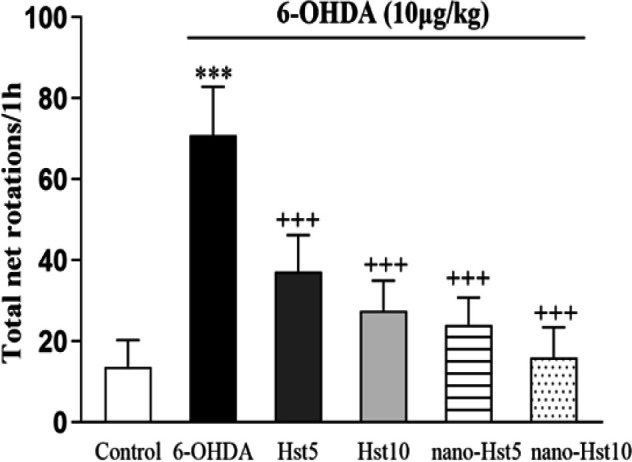
Effect of Hst and nano-Hst on 6-OHDA-induced behavioral changes in apomorphine-induced rotation test. Data are expressed as mean±SD (n=7/group). One-way ANOVA: ***p<0.001 (vs. the control group); +++p<0.001 (vs. the 6-OHDA group). 6-OHDA, 6-hydroxydopamine; Hst, hesperetin.

As shown in Figure 2, the number of contralateral rotations in the Hst 5 (p<0.001), Hst 10 (p<0.001), nano-Hst 5 (p<0.001) and nano-Hst 10 [F (5, 36) = 43.33, p<0.001] groups remarkably reduced compared with the 6-OHDA group. However, there was no remarkable difference between the groups of Hst 5 and Hst 10, and the groups of nano-Hst 5 and nano-Hst 10.

**Effect of Hst and nano-Hst on imbalance behavior induced by 6-OHDA in NBT**

**Figure 3:**
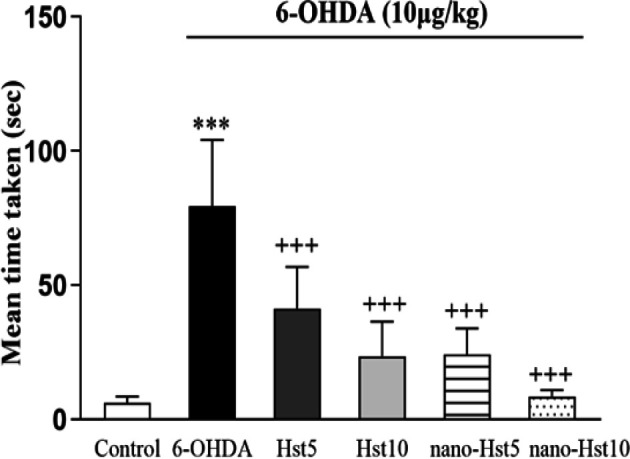
Effect of Hst and nano-Hst on 6-OHDA-induced behavioral changes in narrow beam test. Data are expressed as mean±SD (n =7/group). One-way ANOVA: ***p<0.001 (vs. the control group); +++p<0.001 (vs. the 6-OHDA group). 6-OHDA, 6-hydroxydopamine; Hst, hesperetin.

The NBT revealed that Hst 5 (p<0.001), Hst 10 (p<0.001), nano-Hst 5 (p<0.001) and nano-Hst 10 [F (5, 36) = 27.72, p<0.001] treatments significantly decreased crossing time compared with the 6-OHDA group. However, there was no remarkable difference between groups of Hst 5 and Hst 10, and the groups of nano-Hst 5 and nano-Hst 10 (Figure 3).

**Effect of Hst and nano-Hst on learning and memory deficits** **induced by 6-OHDA in NORT**

**Figure 4:**
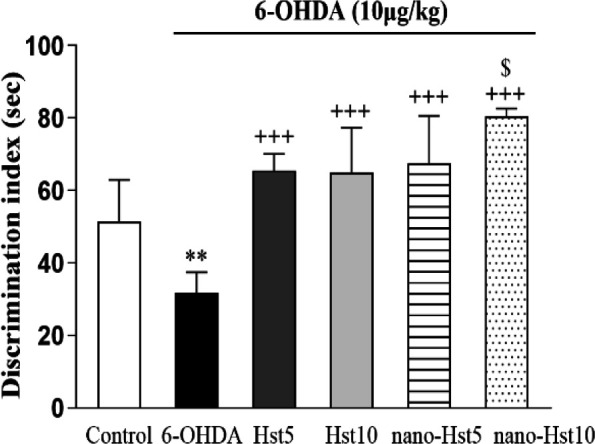
Effect of Hst and nano-Hst on 6-OHDA-induced behavioral changes in novel object recognition test. Data are expressed as mean±SD (n=7/group). One-way ANOVA: **p<0.01 (vs. the control group); +++p<0.001 (vs. the 6-OHDA group); $p<0.05 (vs. the Hst 10 group). 6-OHDA, 6-hydroxydopamine; Hst, hesperetin.

The NORT exhibited that Hst 5 (p<0.001), Hst 10 (p<0.001), nano-Hst 5 (p<0.001) and nano-Hst 10 (p<0.001) treatments significantly increased the discrimination index in comparison with the 6-OHDA group. Moreover, nano-Hst 10 group showed a significant increase [F (5,36)=22.99, p<0.05] in the discrimination index in comparison with Hst 10 group. However, the nano-Hst 10 group did not show remarkable effects on the discrimination index in comparison with the Hst 5 group (Figure 4).

**Effect of Hst and nano-Hst on alterations** **induced by 6-OHDA in MDA and GSH levels**

As shown in Table 2, Hst 5 (p<0.01), Hst 10 (p<0.001), nano-Hst 5 (p<0.001) and nano-Hst 10 (p<0.001) treatments significantly decreased MDA levels in the hippocampus [F(5,36)= 11.64] and striatum [F(5,36)= 8.99] compared with the 6-OHDA group. Also, our results revealed that compared with the 6-OHDA group, nano-Hst 5 (p<0.01) and nano-Hst 10 [F(5,36)= 12.19, p<0.001] treatments in the hippocampus significantly enhanced GSH levels. However, there was no remarkable difference between the Hst 5 and Hst 10 groups, and the nano-Hst 5 and nano-Hst 10 groups in the hippocampus and striatum. 

**Effect of Hst and nano-Hst on alterations induced by 6-OHDA in antioxidant enzymes activity**

As shown in Table 3, compared to the 6-OHDA group, Hst 10 [F (5,36) = 14.63, p<0.001] treatment in the hippocampus and nano-Hst 5 (p<0.01) and nano-Hst 10 (p<0.001) treatments in the hippocampus and striatum significantly increased CAT activity. Also, there was a remarkable difference between the Hst 10 group and nano-Hst 10 group in the striatum levels [F (5,36)= 16.36, p<0.001].

**Table 2:** Effect of Hst and nano-Hst on 6-OHDA-induced alterations in MDA and GSH levels

Groups	MDA (µg/mg protein)	GSH (mg GSH/gr protein)
Hippocampus		
Control	0.11±0.00	0.40±0.06
6-OHDA	0.31±0.03^***^	0.02±0.00^***^
6-OHDA + Hst5	0.19±0.02^++^	0.13±0.03
6-OHDA + Hst10	0.15±0.03^+++^	0.22±0.03
6-OHDA + nano-Hst5	0.15±0.00^+++^	0.29±0.04^++^
6-OHDA + nano-Hst10	0.11±0.00^+++^	0.37±0.04^+++^
Striatum		
Control	0.15±0.00	0.63±0.12
6-OHDA	0.54±0.13^***^	0.03±0.00^**^
6-OHDA + Hst5	0.27±0.05^++^	0.15±0.06
6-OHDA + Hst10	0.13±0.02^+++^	0.17±0.04
6-OHDA + nano-Hst5	0.12±0.01^+++^	0.30±0.11
6-OHDA + nano-Hst10	0.07±0.01^+++^	0.42±0.17

Meanwhile, Hst 10 (p<0.01) and nano-Hst 5 treatment [F (5,36) = 10.94, p<0.01] in the hippocampus, and treatment with nano-Hst 10 in the hippocampus (p<0.001) and striatum [F (5,36) = 5.74, p<0.01] resulted in significantly increased SOD activity in comparison with the 6-OHDA group. In addition, nano-Hst 5 (p<0.01) and nano-Hst 10 [F (5,36) = 8.86, p<0.001] treatments in the hippocampus and striatum resulted in increased GRx activity compared with the 6-OHDA group. Also, there was a remarkable difference (p<0.01) between the Hst 5 group and nano-Hst 5 group in the hippocampus. However, there was no remarkable difference between the Hst 5 and Hst 10 groups, and the nano-Hst 5 and nano-Hst 10 groups in other parameters.

**Effect of Hst and nano-Hst on alterations induced by 6-OHDA in expression levels of** ***CAT*** **,** ***SOD*** **and** ***GRx*** **genes**

**Figure 5:**
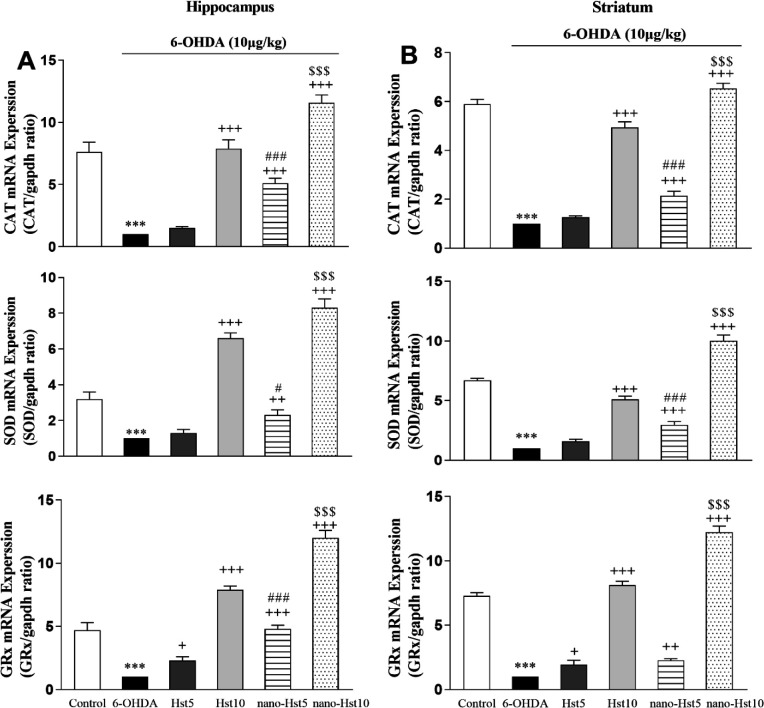
Effect of Hst and nano-Hst on 6-OHDA-induced alterations in expression levels of *CAT*, *SOD* and *GRx* genes in the hippocampus (A) and striatum (B). Data are expressed as mean±SD (n=3/group). One-way ANOVA: ***P<0.001 (vs. the control group); +p<0.05, ++p<0.01, and +++p<0.001 (vs. the 6-OHDA group); #p<0.05 and ###p<0.001 (vs. the Hst 5 group); $$$p<0.001 (vs. the Hst 10 group). 6-OHDA, 6-hydroxydopamine; Hst, hesperetin.

The results of RT-qPCR analysis revealed that Hst 5 treatment significantly increased *GRx* gene expression levels in the hippocampus [F (5,12) = 289.50, p<0.05] and striatum [F (5,12) = 689.80, p<0.05]. Likewise, Hst 10 (p<0.001), nano-Hst 5 (p<0.01) and nano-Hst 10 (p<0.001) treatments significantly increased *CAT* [hippocampus: F (5,12) = 179.94; striatum: F (5,12) = 590.90], *SOD* [hippocampus: F (5,12) = 255.93; striatum: F (5,12)= 476.10] and *GRx* gene expression levels in the hippocampus and striatum. More importantly, comparison of the Hst 5 and Hst 10 groups, nano-Hst 5 (p<0.05) and nano-Hst 10 (p<0.001) groups showed a remarkable difference in gene expression of *GRx*, *CAT* and *SOD* in the hippocampus (Figure 5A) and striatum (Figure 5B).

## Discussion

In the current research, we studied the efficacy of nano-Hst in ameliorating behavioral and biochemical factors in a model of PD induced by 6‐OHDA. Our data showed that the neuroprotective influence of nano-Hst was more marked than Hst at a similar dose.

The experimental model utilized in this study was the 6-OHDA lesioned rat model. Numerous neurochemical changes in PD patients are similar to 6-OHDA-induced neurotoxicity (Hernandez-Baltazar et al., 2017). Our results showed a notable enhancement in contralateral rotation count in 6-OHDA-lesioned rats in AIRT. Furthermore, our assessment in the NBT showed an increase in total time on the beam in 6-OHDA rats, indicating bradykinesia and/or akinesia, in comparison with the control group. 

**Table 3:** Effect of Hst and nano-Hst on 6-OHDA-induced alterations in antioxidant enzymes activity

Groups	CAT (U/mg protein)	SOD (% inhibition)	GRx (U/mg protein)
Hippocampus			
Control	172±12.68	84.93±1.37	188.97±36.10
6-OHDA	54.39±11.07^***^	55.35±1.67^***^	51.41±7.34^**^
6-OHDA + Hst5	88.64±53.55	69.48±3.51	88.62±15.42
6-OHDA + Hst10	182.48±24.67^+++^	72.48±3.72^++^	114.12±23.01
6-OHDA + nano-Hst5	176.67±21.90^+++ ##^	72.95±3.91^++^	193.32±14.35^++ ##^
6-OHDA + nano-Hst10	236.85±21.41^+++^	84.17±4.30^+++^	198.88±18.84^+++^
Striatum			
Control	289.02±46.34	70.16±9.01	257.96±38.23
6-OHDA	62.09±10.24^***^	27.97±1.96^**^	42.66±12.02^***^
6-OHDA + Hst5	108.77±20.88	33.12±6.96	66.28±91.45
6-OHDA + Hst10	158.58±24.12	39.08±7.02	106.74±12.51
6-OHDA + nano-Hst5	217.15±35.04^++^	45.43±9.08	185.39±30.41^++^
6-OHDA + nano-Hst10	397.77±47.78^+++ $$$^	62.82±9.62^++^	214.23±42.80^+++^

As previous studies have shown, prominent motor asymmetry following 6-OHDA injection is due to damage to the nigrostriatal dopaminergic system and consequent reduction in dopamine levels (Antunes et al., 2021). Hst and nano-Hst treatment were able to significantly reduce the dysfunction observed in 6-OHDA rats. In this context, research has pointed out that free radicals play a major role in nigrostriatal lesions induced by 6-OHDA and the protection of dopaminergic neurons in PD (Aguiar et al., 2006). Hence, the survival time of dopaminergic neurons and motor asymmetry in rats can be improved by Hst as a scavenger of free radicals (Kiasalari et al., 2016). In addition to the motor impairments, patients with PD can also show cognitive deficits, specifically learning and memory deficits (He et al., 2018). The hippocampus is one of the areas that take a leading role in learning and memory impairments, and in novelty detection (Chiaravalloti et al., 2014). NORT results showed that 6-OHDA injection causes learning and memory dysfunctions by reducing the discrimination index. This is compatible with our earlier investigation that 6- oxidative damage induced by OHDA is related to the pathogenesis of PD and has a prominent role in learning and memory deficits (Ghaffari et al., 2018). In our study, Hst and nano-Hst protected against 6-OHDA-induced learning and memory deficits as revealed by an increase in the discrimination index in NORT. Previous studies have shown that the mechanism for the learning and memory deficits improving attributed to Hst may be related to its antioxidant properties (Alizadeh Makvandi et al., 2021; Muhammad et al., 2019). One examination also revealed that hesperidin can improve the memory deficit of rats in this model through an increase in dopamine and antioxidants (Antunes et al., 2014). NORT findings also revealed a significant difference between Hst and nano-Hst treatments at dose 10, which is confirmed by our previous study (kheradmand et al., 2018). In agreement with our data, Kesmati et al. exhibited that ZnO and MgO in nanoforms are more able of improving memory performance by increasing the discrimination index in NORT (Kesmati et al., 2020). 

More and more investigations suggest that the survival and maintenance of dopaminergic neurons in PD are related to oxidative stress as an essential and pivotal factor. Free radicals are extremely engaged in the toxicity of nigrostriatal lesions induced by 6-OHDA (Gaba et al., 2019). The enhancement of free radicals caused by the injection of 6-OHDA leads to oxidative damage to membrane lipids and eventually alters the antioxidant enzymes activities (Chandrasekhar et al., 2018). The generation of H_2_O_2_ radicals and 6-OHDA-induced superoxide radicals leads to a decrease in SOD and CAT activities and subsequently 6-OHDA-induced toxicity. The toxicity caused by 6-OHDA may be converted into reactive hydroxyl radicals and subsequently, generate cell injury. The reduction in SOD activity is associated with increases in MDA levels, indicating that these events are preceded by 6-OHDA-induced superoxide radicals generation (Hritcu et al., 2008). In the presence of H_2_O_2_, GSH is oxidized to glutathione disulfide, which is reconverted to GSH by GRx. Since the GRx cycle is inactivated by H_2_O_2_, it reduces the level of GSH, which is often employed as an index of oxidative stress (Kashiwagi et al., 1996). Our findings exhibited that 6-OHDA enhanced lipid peroxidation (estimated in terms of MDA levels), along with decreased GSH levels and antioxidant enzymes gene expression and activity (*CAT*, *SOD* and *GRx*), as we also showed in our previous study (Ghaffari et al., 2018). In line with our results, Baluchnejadmojarad et al. have found out that administration of 6-OHDA is associated with a decline in antioxidant enzymes activities and an enlargement in lipid peroxidation (Baluchnejadmojarad et al., 2017). In this research, Hst at doses of 5 and 10 in some parameters and nano-Hst at doses of 5 and 10 in most parameters were able to mitigate 6-OHDA-induced oxidative stress through induction of a significant increase in gene expression and the activity of antioxidant enzymes which is in line with prior investigations on its antioxidant capacity as well as its ability to alleviate oxidative damage (Samie et al., 2018). In confirmation of our results, Ishola et al. showed that Hst can help maintain the oxidant/ antioxidant balance to a great extent and protect against oxidative stress by inhibiting the production of free radicals (Ishola et al., 2019). Additionally, nano-Hst compared to Hst showed significant effects on some antioxidant parameters in the hippocampus and striatum areas; implying that nano-Hst is a powerful free radical scavenger. According to these results, nano-Hst exhibited stronger effects in improving behavioral disorders, maintaining GSH levels, regulating lipid peroxidation and gene expression and activity of antioxidant enzymes in comparison with Hst in equal doses. The outcome of the current investigation showed that nano-Hst has a better neuroprotective effect than Hst against 6-OHDA-induced lesions, possibly due to its improved bioavailability. In confirmation of our findings, Shete, et al. showed that nano-Hst improves the bioavailability of Hst by increasing its solubility, release rate, penetration into gastric mucosa and intestinal cells. Further, estimation of pharmacokinetic parameters revealed that oral administration of nano-Hst showed a 1.79- and 2.25-fold increase in Cmax and oral bioavailability of Hst, respectively (Shete et al., 2015). In summary, this research provides evidence that treatment with Hst and nano-Hst ameliorated behavioral and biochemical deficits induced by 6-OHDA. The neuroprotective effects of Hst are attributed to its antioxidant activity. Nanonization increased the antioxidant capacity of Hst, which cloud be attributed to its improved oral bioavailability. Thus, it can be summarized that nano-Hst could act as a possible healing agent for PD. Additional research with high-quality study designs is needed to understand more clearly the mechanism implicated in the regulation of antioxidant enzymes expression through the administration of nano-Hst and whether it can be an effective remedy for PD.

## Conflicts of interest

The authors have declared that there is no conflict of interest.
